# Pathophysiology and Imaging Diagnosis of Demyelinating Disorders

**DOI:** 10.3390/brainsci8030044

**Published:** 2018-03-14

**Authors:** Evanthia Bernitsas

**Affiliations:** Wayne State University School of Medicine, Department of Neurology, Detroit, MI 48201, USA; ebernits@med.wayne.edu; Tel.: +1-313-745-4994

The spectrum of “demyelinating disorders” is broad and it includes various disorders with central nervous system (CNS) demyelination, such as multiple sclerosis (MS), Neuromyelitis optica spectrum disorders (NMOSD), transverse myelitis, optic neuritis, acute disseminated encephalomyelitis, overlap and unclassified disorders, with MS being the most common. MS is a complex, multifaceted autoimmune disorder and the most common cause of non-traumatic disability in young adults [1,2]. Considerable research over the recent years has improved our knowledge and led to earlier diagnosis, novel therapeutic strategies, and an overall longer time in the workforce and improved quality of life for MS patients. However, diagnosis and management remain challenging. The disease burden on patients and caregivers is immense. Up until now, there are no FDA-approved remyelinating therapies. MS is still an incurable disease and many questions regarding pathogenesis, diagnosis and treatment remain unanswered.

In this special issue, Zabad et al. review extensively the wide spectrum of demyelinating syndrome, classification, rare and atypical presentations, differential diagnosis and evolution from the first demyelinating episode to the full- blown disease. Serum biomarkers, key imaging findings and management strategies are discussed. Specifically, the presence and significance of aquaporin 4 (AQP-4) and myelin oligodendrocyte glycoprotein (MOG) antibody, myelin basic protein (MBP), glial fibrillary acidic protein (GFAP), S100, MOG, specific cytokines, such as interleukin 6 (IL-6) in the diagnostic evaluation and management is highlighted. This review emphasizes practical points in the “real world” practice that are of valuable assistance to the clinician [3].

Misdiagnosis of MS may occur, especially early in the disease process, as there is a significant number of diseases with similar presentation [4,5]. Over the last decade, the diagnostic accuracy of demyelinating disorders has improved, as advanced diagnostics, especially magnetic resonance imaging (MRI) techniques appear. The development of biomarkers is a necessity, as they have diagnostic, prognostic, and therapeutic value [6]. Using a calibrated functional MRI, Hubbard et al. investigate a new imaging biomarker, the visual-evoked cerebral metabolic rate of oxygen (veCMRO_2_), its contribution in improving diagnostic accuracy and the possibility of being used as a prognostic biomarker in the future in the context of a “gold standard” model of MS diagnostics that combine many relevant factors [7].

The symptoms of MS are non- specific, not always obvious and a number of them cannot be measured objectively. As fatigue is one of the most common, multifactorial, disabling and difficult to treat symptoms, with a severity that can only be evaluated by self-reporting scales, more insight into its pathophysiology and imaging characteristics is needed [8]. The article by Bernitsas et al. sheds light into the pathophysiology of MS-related fatigue and specifically focuses on its volumetric and neural integrity measures in patients with different degrees of pure MS- fatigue and low disability, using advanced MRI technology [9]. 

Comorbidities in MS patients have been extensively studied, as they have a negative impact on the quality of life, management and overall prognosis on MS patients. Comorbidities may delay initiation of disease-modifying treatment, limit therapeutic options and complicate treatment decisions. There is growing evidence that comorbidities may increase relapse rate and disability progression [10,11,12,13,14]. Painful paresthesias are part of the MS symptomatology; however painful sensations can be seen in other conditions co-existing with MS and may lead to diagnostic confusion. The review article by Purvis et al. focuses on the concurrent presence of cervical spondylotic myelopathy in MS patients that is commonly seen in everyday clinical practice and evaluates the results of decompressive surgery on pain management and quality of life in this population. The need for a comprehensive approach and multidisciplinary collaboration is emphasized [15].

Pathophysiology of demyelinating disorders is complex and not very well understood. The contribution of B-lymphocytes has been increasingly acknowledged, in addition to the traditional view regarding the role of T-lymphocytes in demyelinating pathophysiology. There are various B and T subsets, as well as different cell populations that are key players in the immune response and their involvement has been further investigated. 

In this special issue, three review articles discuss MS pathogenesis and address old and new knowledge. In a very comprehensive review by Dargahi et al., the pathophysiology of MS is explained and the role of specific cells, including T and B-lymphocytes and their subsets, macrophages, microglia, natural killer and dendritic cells, in the pathogenesis of demyelination is further analyzed [16]. Kinzel et al. review the role of humoral immunity in demyelinating disorders and further explore the role of peripheral CNS-specific antibodies in initiating a cascade of events that lead to CNS demyelination [17]. As MS encompasses both an inflammatory and a neurodegenerative component, with neurodegeneration being more prominent later in the disease course and especially during the progressive stage and associated with disability, Salapa et al. discuss the role of neuronal and axonal damage in MS, emphasize the multifactorial nature of neurodegeneration and summarize potential mechanisms that contribute to neuro-axonal injury. [18].

A new, deep insight into MS pathogenesis may promote novel neuroprotective and remyelinating therapeutic strategies. The review by Bose focuses on a very specific population of cells in MS pathophysiology, the T, B and resident memory cells, their role in MS pathophysiology, the effect of the disease modifying agents on this cell population and their potential of being a therapeutic target [19]. Lisak and Benjamins review melanocortins and their receptors (MCR), and analyze the direct effect of melanocortins on the CNS (neurons and glia) as well as their effect on the immune cells in the periphery. The role of adrenocorticotropic hormone (ACTH) in treating MS relapses is discussed and comparative efficacy results between ACTH and intravenous steroids from clinical trials are presented. In this review article, future research targets are explored and the potential for developing innovative neuroprotective therapies involving MCR agonists is highlighted [20]. As there is growing interest in cell-based therapeutic strategies for MS [21], more research is needed. Emerging immunotherapeutic approaches, such as stem cells, nanoparticles, mannan, DNA vaccines, altered peptide ligands and cyclic peptides, are presented by Dargahi et al. [16], after reviewing current and approved disease-modifying agents. 
